# Study on time effect of residual pressure and bearing characteristics of jacked pile in saturated silt foundation

**DOI:** 10.1038/s41598-022-25179-1

**Published:** 2022-11-28

**Authors:** Yong-Zhi Jiu, Xiang-Yu Zhang, Yan-zhi Zhu, Zhen Zhang, Guang-hui Tian

**Affiliations:** School of Civil Engineering and Architecture, Zhongyuan University of Technology, Zhengzhou, 450007 China

**Keywords:** Engineering, Civil engineering

## Abstract

The history of stress in soil mass and pile surface roughness significantly impacts the time effect of residual pressure at the pile end and bearing characteristics of the jacked pile. In this study, the impacts of soil over-consolidation ratio and pile surface roughness on the time effect of residual pressure and bearing characteristics of jacked pile end in saturated silt foundation are explored. Through the independently developed model test device for the vertical bearing characteristics of jacked pile, the driving of jacked pile with different pile surface roughness and static load tests at different resting phases are carried out on saturated silt foundations with different over-consolidation ratios. The model box is cylindrical in shape with a size of 40 cm × 48 cm (inner diameter × height) and is made of transparent tempered glass. The results show that: the increase in surface roughness of jacked pile in saturated silt foundation causes not only the increase in the pile side friction but also the increase in the pile end resistance during the static pressure sinking pile; the change laws on the residual pressure of pile end and limit friction resistance of pile side for jacked pile in saturated silt foundation vary with over-consolidation ratio of soil mass and the pile surface roughness.

## Introduction

Due to the advantages of rapid construction progress and a high degree of civilization^1–3^, jacked pile are more and more widely adopted, especially in urban areas with dense population or areas with special requirements for vibration. During the driving of jacked pile in saturated soil foundations, due to the soil squeezing effect, the original stress state of the foundation is disturbed and excess pore-water pressure is generated. Once the pile driving is completed, the excess pore-water pressure generated by the pile driving gradually dissipates with time, and the effective stress of soil around the pile increases accordingly. The strength and stiffness of soil around the pile gradually recover and improve with the extension of the resting phase, making the bearing characteristics of the jacked pile present an obvious time effect^4–7^. The research on the bearing characteristics of jacked piles and the time effect of bearing characteristics of the jacked piles has always been the area of concern for the researchers^8–13^. Due to the complex changes in the mechanical properties of the soil around the pile during the driving of the jacked pile and the load-bearing process, the theoretical analysis model for the time effect of the bearing characteristics of the jacked pile is often simplified to a larger extent. It significantly affects the comparability between theoretical analysis results and measured results. Therefore, experimental investigations are needed to explore the time effect of vertical bearing characteristics of jacked pile.

Because of the time effect of the bearing characteristics of jacked pile, Gwizdała^14^, Skov and Svinkin^15^, Jardine et al.^16^ and Zhang et al.^17^ conducted a large number of theoretical derivations and experimental studies through the in-situ test method. Many researchers investigated the time effect of ultimate bearing capacity of jacked pile in saturated soil foundation and explored the internal mechanisms and factors that affect the time effect of bearing characteristics of jacked pile. Roy et al.^18^, York et al.^19^, Axelsson^20^, Long et al.^21^ Gwizdała and Więcławski^22^ conducted static load tests on different resting phases after completion of driving of the jacked pile. Zhang et al.^23^ conducted interval re-pressing tests on jacked pipe pile in saturated soft clay foundation and found that the re-pressing start pile pressure increased by more than 2.5 times 25 days after pile driving. A higher rate in the early stage and a lower rate in the latter stage were observed. It was argued that the interval re-pressing pressure of jacked pile could reasonably replace the ultimate bearing capacity of a single pile. Dong and Guo^24^ carried out static load testing on jacked PHC pipe piles based on interval re-pressing tests. The results indicated that the re-pressing value and growth rate are related to the properties of the soil layer. Under the same soil layer properties, the growth value and the growth rate of re-pressing stress were the same. Kou et al.^25^ and Tang et al.^26^ buried sensing devices in the pile body and determined the time-dependent changes in the bearing capacity, pile end resistance, and side friction resistance of PHC open piles through the interval re-pressing tests. The results showed that the ultimate bearing capacity of the pile increases in a logarithmic manner within a certain time range after the completion of pile driving, and the growth of bearing capacity mainly depended on the side friction.

Some researchers have also conducted field test research and analysis on the time effect of bearing characteristics of jacked pile, focusing on the time effect of side friction of jacked pile. Zhang et al.^13^ studied the relationship between the instantaneous terminal pressure and the ultimate bearing capacity of jacked pile from aspects of the jacked pile driving process and the time effect of bearing capacity and proposed the concepts of side resistance degeneration and time effect coefficients. Wang and Zhang^27^ demonstrated through the interval re-pressing test of jacked pipe piles in saturated soft clay foundations that the re-pressing start-up pressing pile force increased significantly, which increased faster in the early stage and slower in the later stage. By analyzing the aging mechanism for jacked pipe pile, it was shown that the increase of the single-pile bearing capacity of jacked pile with time was primarily due to the increase in the pile side resistance. The change in pile end resistance contributed less to the time effect of bearing capacity. Hu et al.^28^ conducted in-situ tests on 300 mm diameter piles. The results showed that when the pile shaft friction was dry friction, there was almost no increase in the time-dependent capacity. However, when the pile shaft friction was lubricated friction, then the time-dependent effect of the bearing capacity was much pronounced.

Once the driving of the jacked pile is completed and the pressing pile force is removed from the pile top, the elastic compression deformation of the pile body is partially recovered due to the confinement effect of soil around the pile, and the relevant stress detained on the pile is the residual stress of construction^29^, including residual stress of pile body, residual unit friction resistance of pile side, and residual stress of pile end. Their properties are closely related to the soil layer in pile driving^30^. The residual stress in jacked pile driving construction has an important effect on the bearing characteristics of the pile foundations^31,32^. Kou et al.^25^, Yu et al.^29^, and Zhang and Wang^33^ experimentally investigated the time effect of residual stresses of the jacked pile; however, rather contradictory findings were presented.

Field tests are usually conducted to determine the time effect of bearing characteristics of jacked pile. Different sites significantly differ in soil mass category and the history of stress in the soil mass. Thus, accurately controlling the stress state of in-situ soil mass is challenging. Under the influence of various on-site conditions and objective factors, the number of test groups is often smaller, so it is difficult to systematically study the time effect of bearing characteristics of jacked pile through field tests. Therefore, the model test has become an indispensable tool for research on the time effect of bearing characteristics of jacked pile. However, the laboratory model tests on jacked pile mainly focus on the penetration mechanism of jacked pile. There are fewer laboratory model tests on the time effect of residual stress and bearing characteristics of jacked pile.

The variation trends of residual stress, pile side ultimate friction, and bearing characteristics of jacked piles with time obtained from existing research are not consistent, and many researchers have tried to study and analyze it from different aspects. However, there are fewer studies on the effects of the over-consolidation ratio of soil and pile surface roughness on the time effect of residual pressure, pile side ultimate friction, and bearing characteristics of jacked piles. In this paper, silty clay from the site of a residential project in Zhengzhou, China, was used. Through the independently developed model test device for the vertical bearing characteristics of jacked pile^34^, an experimental investigation was carried out on the time effect of residual pressure at the pile end and bearing characteristics of jacked pile in saturated silt soil foundations. Also, the effects of the over-consolidation ratio of soil and pile surface roughness on the time effect of residual pressure at the pile end, pile side ultimate friction, and bearing characteristics of jacked piles are explored.

## Overview of the model test

### Model test system

**Figure 1:**
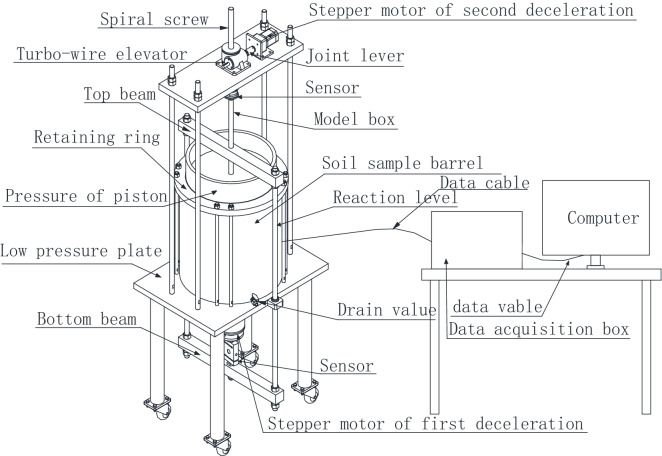
Overall schematic diagram for the model device.

**Figure 2:**
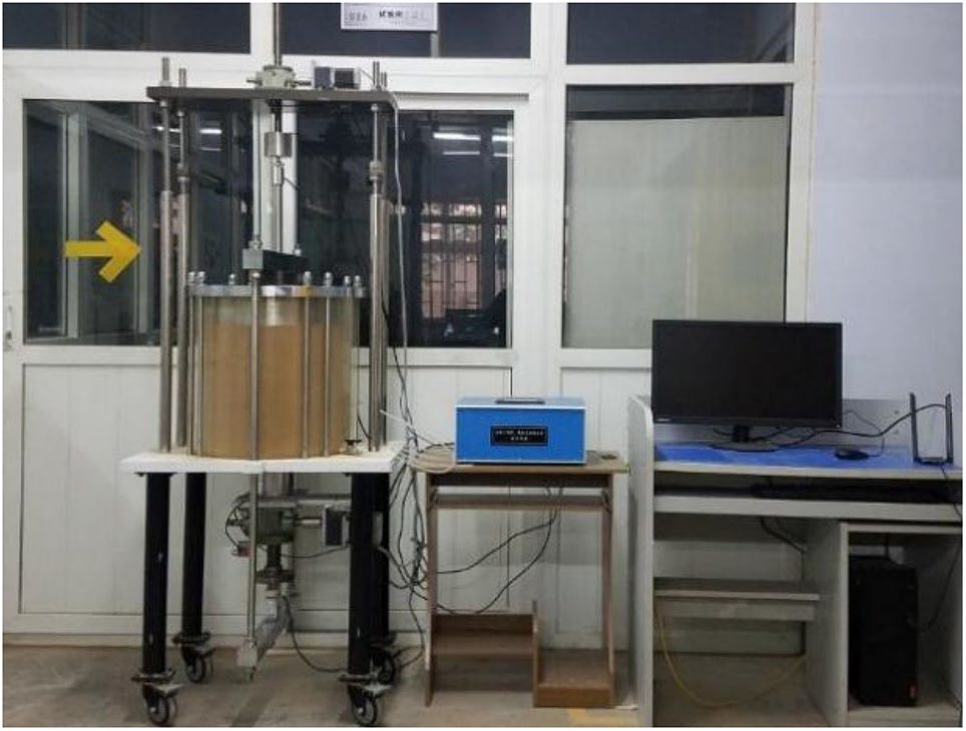
Model test device setup.

Figures 1 and 2 show the model test system used in the test^34^. The model box is cylindrical in shape with a size of 40 cm × 48 cm (inner diameter × height) and is made of transparent tempered glass. The model test system consists of a soil pressure loading system, a model pile loading system, a soil vacuum saturation system, a model box, a model pile, and a control and data acquisition system. The soil in the model box is loaded and unloaded by the soil pressure loading system to simulate different loading paths of soil mass. Therefore, the soil mass in the model box and the actual project have the same stress state and stress history.

### Soil sample preparation

**Figure 3:**
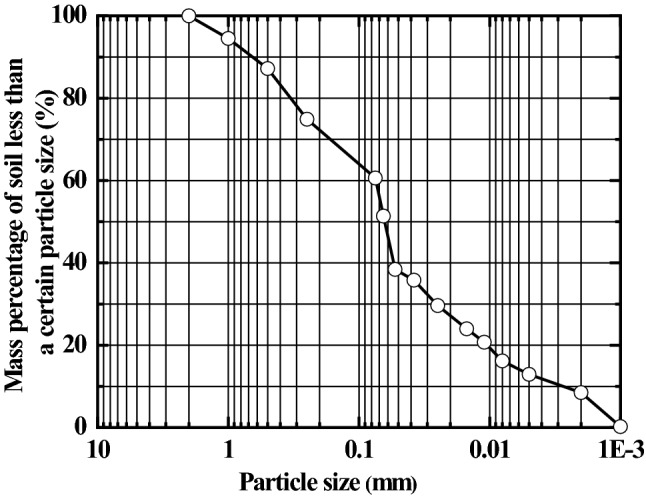
Grain-size distribution curve of testing soil.

The soil used in the laboratory model test was taken from an engineering site in Zhengzhou, China. After drying, the soil was smashed with a rubber hammer and sieved through a sieve of 2 mm size. The grain-size distribution curve of the soil is shown in Fig. 3. The silt with a particle size of less than 2 mm was selected to prepare the silt mud with a moisture content of 100%, which is fully stirred. The basic physical parameters for soil are shown in Table 1. It can be seen from Fig. 3 and Table 1 that the particles having a size greater than 0.075 mm account for 39.4% of the total mass. Also, the plasticity index is 8.8. The soil used in the test is silt, as per the classification of Code for Investigation of Geotechnical Engineering (GB50021-2001, 2009 Edition)^35^.

**Table 1:** Basic physical parameters of soil.

Plastic limit %	Liquid limit %	*K*_0_	$\lambda$	$\kappa$	$M$
14	22.8	0.6	0.045	0.00453	1.09

### Model box loading

After standing for 12 h, the mixed silt mud was put into the model box of the test device in stages. The vacuum method was used to saturate the silt mud in the model box to ensure that the silt mud had a higher saturation degree^34^. The steps for model box loading are as follows.

1. Fine sand with a thickness of about 3 cm was laid at the bottom of the model box, and then distilled water was added to the model box. The water surface was kept 1 cm higher than the fine sand. Finally, vacuum saturation was performed, and after the internal pressure of the model box reached − 95 kPa atmospheric pressure, it continued to vacuum for two hours.
2. A layer of filter paper was placed on the fine sand surface, and silt mud was put at the height of about 10 cm into the model box each time. Vacuum saturation was then performed for two hours. Close the air extraction valve and vacuum pump until the last vacuum saturation is completed. The vacuum degree was kept above − 95 kPa in the model box for 24 h to ensure that the silt mud in the model box was fully saturated.
3. After the soil sample was loaded and vacuum saturation was completed, a layer of filter paper was laid on the top surface of the soil sample, and a layer of fine sand with a thickness of about 1 cm was laid on the filter paper. After the fine sand was allowed to stand for 12 h, a pressure piston was covered, and the upper and lower beams of the test device were installed.

### Model pile

**Figure 4:**
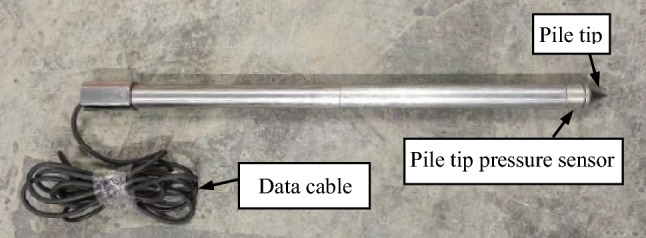
Smooth pile (with the pile surface untreated).

**Figure 5:**
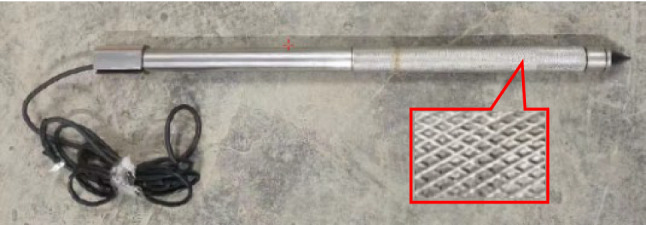
Rough pile (with the pile surface subjected to roughening (scoring) treatment).

In this paper, tests are systematically carried out on the time effect of pile tip residual pressure and bearing characteristics of jacked pile with two kinds of pile surface roughness (smooth pile with untreated pile surface and rough pile with the pile surface subjected to roughening (scouring) treatment, as shown in Figs. 4 and 5) in saturated silt foundations.

The model pile (diameter of 2.5 cm) comprises of pile tip, force sensor, and steel pipe with a length of 40 cm (Figs. 4 and 5). The material of the model pile is 304 stainless steel, with an elastic modulus of 200 GPa and Poisson’s ratio of 0.3. The force sensor at the pile tip has a measurement range of 0–3 kN, a resolution ratio of 1 N, and an accuracy of 0.3% F.S. The pile end resistance is measured, fed back, and recorded in real time by the force sensor.

### Test scheme

In order to study the influence of the pile surface roughness and the over-consolidation ratio of foundation soil on the residual pressure at the pile end and bearing characteristics of jacked pile in saturated silt foundation, in this paper, the laboratory model test was carried out on the time effect of pile driving and bearing characteristics of jacked pile in saturated silt foundation. Table 2 shows the test scheme. Li and Liu^36^ found in the field test that, compared with the first load test, the repeated pressure test on the same pile reduces the settlement under the same load, but the ultimate bearing capacity does not change. It was pointed out that the repeated pressure test on the same pile with different resting phases could be used to study the time effect of bearing characteristics of the pile. Jardine et al.^16^ found the same phenomenon in the test. It shows that it is reasonable to study the time effect of pile bearing characteristics by using the same pile for repeated pressure tests with different resting phases. The *Technical Specifications for the Testing of Building Foundation Pile* (JGJ106-2014)^37^ stipulates that the resting phase for the test of bearing capacity of foundation piles in silt soil foundation is 10 days, so the longest resting phase in this test is 10 days.

**Table 2:** Test scheme.

No.	Soil consolidation pressure/kPa	Type of pile surface	Unloading vertical pressure/kPa	Consolidation type of foundation soil	Jacked pile driving	Static load test
1	50 kPa	Smooth pile	–	Normally consolidated soil	After the consolidation is completed, the model pile driving test is carried out	Single-pile static load test is carried out on Day 2, Day 9, and Day 19 after pile driving (the resting phases are 2 days, 7 days, and 10 days, respectively)
2	50 kPa	Rough pile				
3	150 kPa	Smooth pile	50 kPa	Over-consolidated soil	Model pile driving test is carried out after unloading becomes stable	
4	150 kPa	Rough pile				

The pile sinking and vertical loading tests were carried out on the model pile using the second deceleration stepping motor to drive the turbine screw to rise and fall as well as apply vertical load to the model pile, as shown in Fig. 1. The pile sinking rate has a significant impact on pile sinking and bearing characteristics of the jacked pile^38–40^. In this paper, the impact of the pile sinking rate is not considered in the model test, and the pile sinking rate is 2 mm/min.

## Analysis of test results

### Soil consolidation and jacked pile driving test

**Figure 6:**
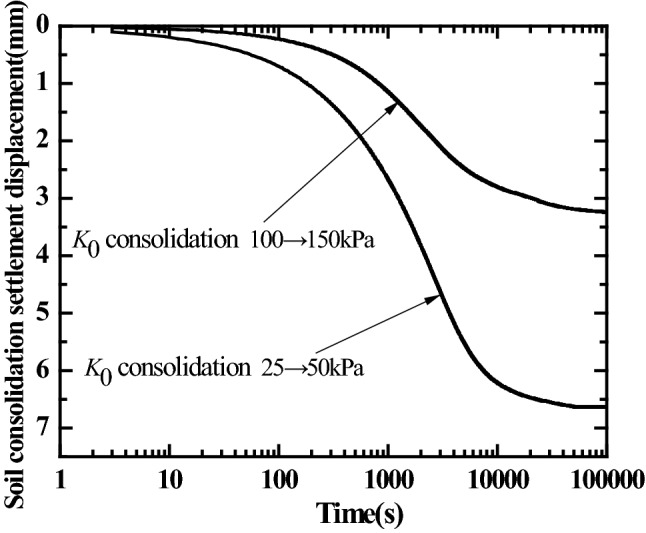
Curves for settlement displacement of soil *K*_0_ consolidation with time.

The method of step loading (2 kPa, 12.5 kPa, 25 kPa, 50 kPa, 100 kPa, 150 kPa) was adopted for the saturated silt consolidation loading in the model box. Figure 6 shows the variation curves for consolidation settlement displacement of soil mass in the model box with time under the consolidation pressure under the maximum vertical consolidation pressure of 50 kPa and 150 kPa, respectively. It can be seen that the primary consolidation has been completed for the soil in the model box under the corresponding maximum consolidation pressure.

**Figure 7:**
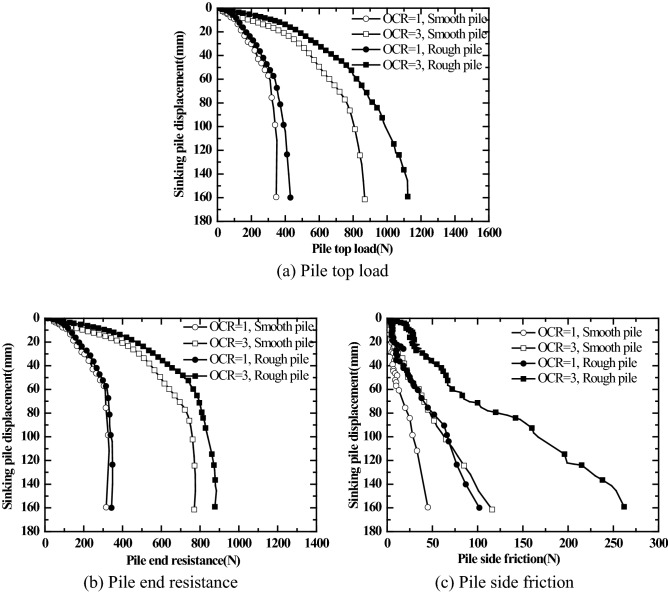
Relation curves for pile top (pile end resistance, pile side friction)—Pile Sinking Displacement.

After the consolidation of the saturated silt in the model box was completed, jacked pile driving test was conducted on the model piles with different surface roughness. Figure 7 shows the relation curves for the variation of pile driving resistance, pile end resistance, and pile side friction with the pile driving displacement during pile driving. The skin friction of the pile is calculated by Eq. (1).

1 $$ P_{s} { = }P_{t} - P_{e} , $$

where, $P_{s}$ is the pile side friction; $P_{t}$ is the pile top load; $P_{e}$ is the pile end resistance.

It can be seen from Fig. 7 that when the pile driving displacement is small, the pile side friction is small due to the small contact area between the pile surface and soil mass. Therefore, the load of jacked pile driving is primarily borne by pile end resistance. With the increase in the pile driving displacement of jacked pile, the contact area between the pile surface and soil mass increases, and the pile side friction gradually increases. As the pile driving displacement of jacked pile continues to increase, the pile end resistance gradually reaches the limit value and keeps stable. In this case, the increase of the pile driving a load of jacked pile is mainly caused by the increase of pile side friction.

It can be seen in Fig. 7 that the pile driving resistance, pile end resistance, and pile side friction of jacked pile in the over-consolidated (OCR = 3) saturated silt foundation during the pile driving process are all greater than those for jacked pile in normally consolidated saturated silt foundation under the same overburden pressure (50 kPa). It can also be seen from Fig. 7 that under the same overburden pressure (50 kPa), whether it is in a normally consolidated saturated silt foundation or over-consolidated (OCR = 3) saturated silt foundation, the pile driving resistance and the pile side friction of rough piles are greater than those of smooth piles. According to Fig. 7b, the pile end resistance of rough piles is greater than that of smooth piles during the pile driving of jacked pile, i.e., the pile surface roughness affects the pile end resistance during the pile driving of jacked pile—the greater the pile surface roughness, the greater the pile end resistance.

### Analysis of time effect analysis of residual pressure at pile end

**Figure 8:**
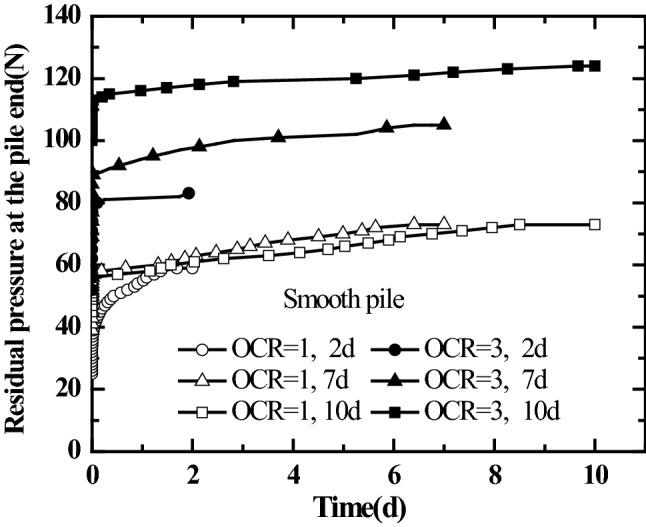
Curves for residual pressure at the smooth pile end varying with time.

**Figure 9:**
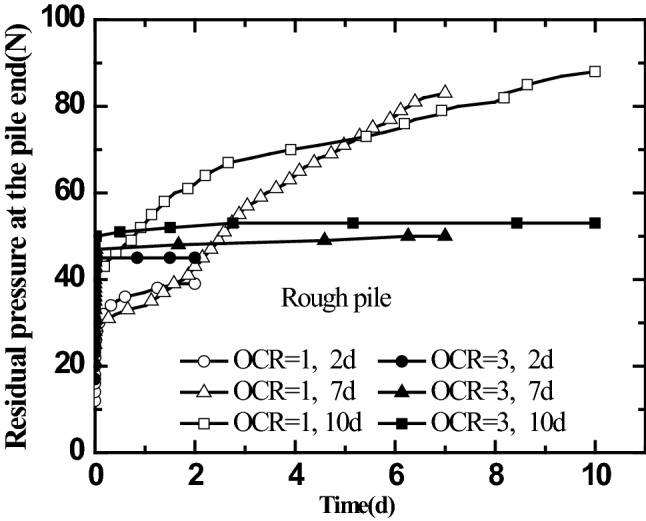
Curves for residual pressure at the rough pile end varying with time.

The residual stress in jacked pile construction impacts the bearing characteristics of pile foundation^31,32^. Kou et al.^25^, Yu et al.^29^, Zhang and Wang^33^, experimentally investigated the time effect of residual stress in jacked pile. However, the conclusions were not the same. It was argued that since the impacts of pile surface roughness and the history of stress in foundation soil on the time effect of residual stress in jacked pile were not considered in the above test procedures, thus different conclusions were obtained. Therefore, a laboratory model test was carried out on the time effect of residual pressure at the pile end of jacked pile with different pile surface roughness in saturated silt foundation with different over-consolidation ratios (OCR = 1, 3). The real-time measurement was performed on the changes in residual pressure at the pile end of jacked pile with time during the resting phase of 2 days. After the jacked pile driving was completed and the pile top load was removed, the resting phase of 7 days after the first load test, the resting phase of 10 days after the second load test, and the variation curves for residual pressure at the pile end of jacked pile with different surface roughness in the saturated silt foundation with different over-consolidation ratios over time were obtained. The results are shown in Figs. 8 and 9.

As shown in Figs. 8 and 9, after the resting phase begins, the residual pressure at the pile end first increases rapidly within a short period. It then enters a relatively slowly increasing (or stable) stage. Moreover, the residual pressure at the end of the jacked pile increases with the number of static load tests, i.e., the static load tests cause the increase of the residual pressure at the pile end of the jacked pile.

It can be seen from Fig. 8 that the residual pressure at the pile end in the over-consolidated saturated silt foundation (the vertical consolidation pressure of soil mass is 50 kPa, OCR = 3) is greater than that in normally consolidated soil mass (the vertical consolidation pressure of soil mass is 50 kPa, OCR = 1). Also, from Fig. 9, it is clear that the change in residual pressure at the pile end of the rough jacked pile with time presents different trends from that of the smooth jacked pile. After increasing rapidly within a short period, the residual pressure at the pile end of the rough jacked pile in the over-consolidated saturated silt foundation enters a stable stage (the residual pressure value remains unchanged). However, the residual pressure at the pile end of the rough jacked pile in the normally consolidated saturated silt foundation increases at a lower rate after the rapid growth in the early stage. However, it still increases over time, and its value gradually exceeds the residual pressure at the pile end of rough jacked pile in the over-consolidated saturated silt foundation.

As seen from Figs. 8 and 9, after rapid growth in the early stage, the residual pressure at the pile ends of the two types of jacked pile (smooth piles and rough piles) with different roughness in over-consolidated saturated silt foundation quickly enters a stable state (the residual pressure at the pile end changes less or does not change) after the resting phase begins, and the residual pressure at the end of the smooth pile is significantly greater than that of the rough pile. It can be seen from Figs. 8 and 9 that for jacked pile with different pile surface roughness in the normally consolidated saturated silt foundation, the residual pressure at the pile end presents similar variation laws with the resting phase after the completion of pile driving. After the resting phase begins, the residual pressure at the pile end of the jacked pile begins to increase gradually with time at a lower rate after the rapid growth in the early stage. The rapid increase of the residual pressure at the pile end in the early stage is due to the increase of the strength of the soil at the pile end caused by the dissipation of the excess pore water pressure at the pile end. The increase of residual pressure at the pile end in the later period (after 2 days) should be attributed to the aging effect of the soil at the pile end.

### Analysis of time effect of bearing characteristics of jacked pile

**Figure 10:**
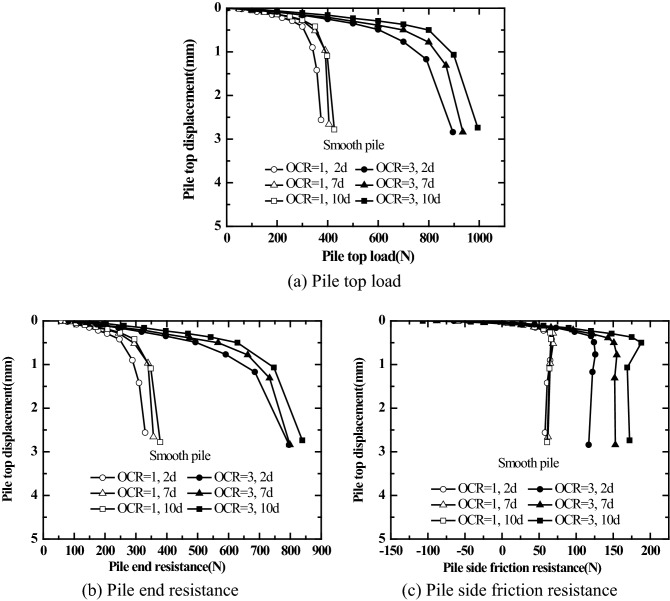
Pile top load (Pile end resistance, Pile side friction resistance)-pile top displacement relations for smooth piles under different over-consolidation ratios.

**Figure11:**
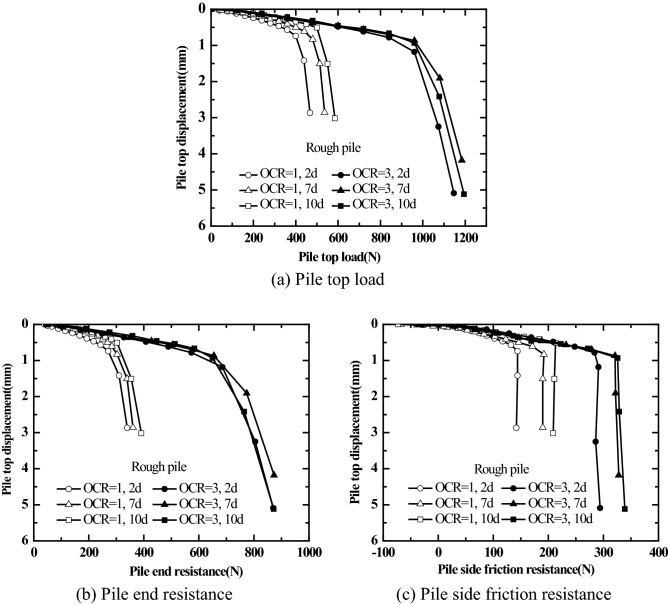
Pile top load (Pile end resistance, Pile side friction resistance)-pile top displacement relations for rough piles under different over-consolidation ratios.

Figures 10 and 11 show the static load test results obtained at different resting phases of jacked pile with different surface roughness in saturated silt foundation for different over-consolidation ratios (the vertical consolidation pressure of soil mass is 50 kPa, OCR = 1, 3). The first static load test was carried out at the resting phase of 2d after pile driving; the second load test was carried out at the resting phase of 7 days after the first load test was completed; the third load test was carried out at the resting phase of 10 days after the second load test was completed.

As shown in Figs. 10 and 11, the pile top stiffness, ultimate bearing capacity, ultimate end resistance, and ultimate pile side friction resistance of jacked pile (smooth piles and rough piles) in the over-consolidated saturated silt foundation are all greater than those in the normally consolidated saturated silt foundation at different resting phases (2 days, 7 days, and 10 days). For the overburden pressure of soil mass of 50 kPa and OCR = 1, 3, the ultimate pile side friction resistance of rough jacked pile is significantly greater than that of smooth piles. It holds good regardless of normally consolidated saturated silt foundation or over-consolidated saturated silt foundation.

It can be seen from Figs. 10a and 11a that the load-settlement behavior does not show a significant change in vales even after there is a significant change in residual pressure (as shown in Figs. 8 and 9) before the load test. Thus, the residual pressure at the pile tip has no significant effect on the bearing characteristics of the pile top.

**Figure 12:**
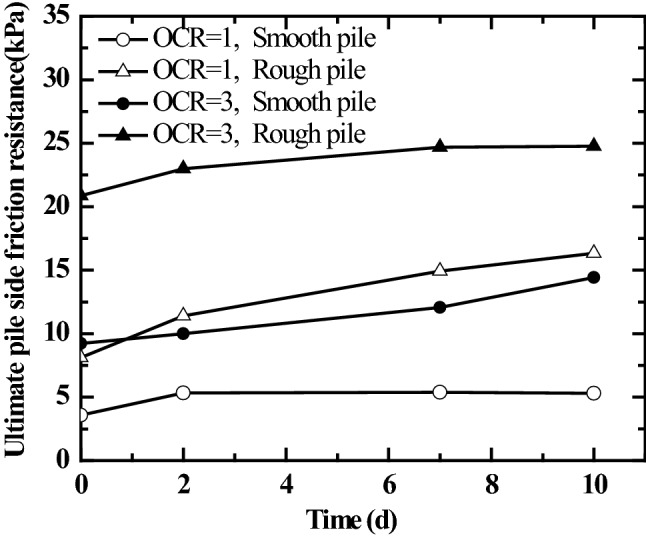
Curves of the ultimate pile side friction resistance of jacked pile varying with the resting phase.

Figure 12 shows the curves of ultimate pile side friction resistance of jacked pile varying with the resting phase. It can be seen from the figure that the time-effect variation trend of ultimate friction resistance of jacked pile in saturated silt foundation varies with the over-consolidation ratio of soil mass and pile surface roughness. For the ultimate pile side friction resistance of smooth jacked pile in the normally consolidated saturated silt (the vertical consolidation pressure of soil mass is 50 kPa, OCR = 1), it reaches the limiting value at the resting phase of 2d after the completion of the jacked pile driving and then remains unchanged, the limit value of pile side friction increased by 48% compared with that of pile sinking. The ultimate pile side friction resistance of rough jacked pile in the over-consolidated saturated silt foundation (the vertical consolidation pressure of soil mass is 50 kPa, OCR = 3) reaches the limiting value at the resting phase of 7d after the completion of the jacked pile driving and then remains unchanged, the limit value of pile side friction increased by 18.4% compared to that of pile sinking. The ultimate pile side friction resistance of rough jacked pile in the normally consolidated saturated silt foundation and that of smooth jacked pile in the over-consolidated saturated silt foundation always maintain an increasing trend within the resting phase of 10 days (the longest resting phase in this test is 10 days), the ultimate pile side friction resistance of pile at the resting phase of 10 days increased by 101% and 56%, respectively, compared with that of pile sinking.

Tomlinson^41^ showed that the ultimate pile side friction resistance is related to the undrained shear strength of soil mass on the pile side. Equation (2) was presented for calculating the ultimate pile side friction resistance.

2 $$ \tau_{f} = \alpha c_{u} , $$

where $\tau_{f}$ is the pile skin friction capacity, $\alpha$ is the pile side friction coefficient, $c_{u}$ is the undrained shear strength of soil around pile.

Coduto^42^ presented the relationship between α and *C*_*u*_ as given in Eq. (3).

3 $$ \alpha = \left\{ \begin{gathered} 1\quad {(}c_{u} \le 32\,{\text{kPa)}} \hfill \\ 0.35 + 170c_{u}^{ - 1.6} \quad {(}c_{u} > 32\,{\text{kPa)}}{.} \hfill \\ \end{gathered} \right. $$

API^43^ suggested that the value for *α* could be determined using Eq. (4).

4 $$ \alpha = \left\{ {\begin{array}{*{20}c} {0.5\left( {\frac{{\sigma_{v0}^{{}} }}{{c_{u} }}} \right)^{0.5} } & {(c_{u} /\sigma_{v0}^{\prime } \le 1.0)} \\ {0.5\left( {\frac{{\sigma_{v0}^{\prime } }}{{c_{u} }}} \right)0.25} & {(c_{u} /\sigma_{v0}^{\prime } \le 1.0)} \\ \end{array} ,} \right. $$

where $\sigma^{\prime}_{v0}$ is the vertical effective stress of the soil around the pile.

This paper used the GDS stress path triaxial apparatus to carry out the stress path triaxial test on the soil in the model test. The undrained shear strength of saturated silt under the stress path condition that is the same as that of soil in the model box is obtained. In addition, Eqs. (3) and (4) are used to calculate the ultimate pile side friction resistance under the model test conditions in this paper, as shown in Table 3.

**Table 3:** Test values and predicted values for ultimate pile side friction resistance (overburden pressure of soil 50 kPa).

Type of model pile	*OCR*	*c*_u_ (kPa)	$\tau_{f}$ (kPa)		
			Resting phase 10 days	API (1993)	Coduto (1994)
Smooth pile	1	24.05	5.31	17.33	24.05
Rough pile			16.32		
Smooth pile	3	51.51	14.41	25.56	33.99
Rough pile			24.76		

It can be seen from Table 3 that the calculation results through the formula proposed by API^43^ are closer to the ultimate pile side friction resistance of rough jacked piles in this paper at the resting phase of 10 days. (The *Technical Specifications for the Testing of Building Foundation Pile* (JGJ106-2014)^37^ stipulates that the resting phase for the test of bearing capacity of foundation piles in silt soil foundation is 10 days). The calculation results through the formula proposed by Coduto^42^ are significantly greater than the test results in this paper.

## Conclusion

In this paper, the impacts of the stress history of saturated silt foundation and pile surface roughness on the time effect of residual pressure at the pile end and bearing characteristics of jacked pile are studied.

The following main conclusions are drawn from the study.

1. Under the same overburden pressure, the greater the over-consolidation ratio of saturated silt foundation, the greater the pile driving resistance, the pile end resistance, and the pile side friction resistance during the pile driving process of jacked pile. The increase in pile surface roughness causes the increase in pile side friction resistance and pile end resistance during pile driving of jacked pile.
2. The residual pressure at the jacked pile end in saturated silt foundation is related to pile surface roughness and the history of soil stress. For the piles with smaller surface roughness (smooth piles), under the same overburden pressure, the greater the over-consolidation ratio of soil, the greater the residual pressure at the pile end. In the normally consolidated saturated silt foundations, the variation trends on the residual pressure at the pile ends of jacked pile with different surface roughness with the resting phase are similar after the completion of the pile driving. Thus, after the resting phase begins, the residual pressure at the pile end of the jacked pile increases gradually at a lower rate after the rapid growth in the early stage. In the over-consolidated saturated silt foundation, the residual pressure at the pile end rapidly increases after the beginning of the resting phase. It then rapidly enters a stable stage (the residual pressure at the pile end changes less or does not change). The residual pressure at the end of smooth piles is significantly greater than that of rough piles.
3. The variation laws on the time effect of the ultimate pile side friction resistance of jacked pile in saturated silt foundation vary with the over-consolidation ratio of soil and pile surface roughness. The ultimate pile side friction resistance of smooth jacked piles in normally consolidated saturated silt remains unchanged after reaching the limit value at the resting phase of 2 days after the completion of jacked pile driving. The limit value of pile side friction increased by 48% compared to pile sinking. The ultimate pile side friction resistance of rough jacked pile in over-consolidated saturated silt foundation remains unchanged after reaching the limit value at the resting phase of 7 days after the completion of jacked pile driving. The limit value of pile side friction increased by 18.4% compared to pile sinking. The ultimate pile side friction resistance of rough jacked piles in normally consolidated saturated silt foundation and that of smooth jacked piles in over-consolidated saturated silt foundation both maintain an increasing trend within the resting phase of 10 days (the longest resting phase in the test in this paper is 10 days). The ultimate pile side friction resistance of pile at the resting phase of 10 days increased by 101% and 56%, respectively, compared with that of pile sinking.
4. Due to the small size of the model test, the conclusions summarized based on the model tests in this paper need to be further verified in the actual project.

## Data Availability

The datasets used in the current study are available from the corresponding author upon reasonable request.
